# Characterizing the Assessment and Management of Vitamin D Levels in Patients with Osteoporosis in Clinical Practice: A Chart Review Initiative

**DOI:** 10.1155/2015/312952

**Published:** 2015-01-29

**Authors:** Jonathan D. Adachi, Jacques P. Brown, George Ioannidis

**Affiliations:** ^1^Charlton Medical Centre, Department of Medicine, McMaster University, 25 Charlton Avenue E., Suite 501, Hamilton, ON, Canada L8N 1Y2; ^2^Groupe de Recherche en Rhumatologie et Maladies Osseuses, Centre de Recherche du Centre Hospitalier Universitaire de Québec, Laval University, 2705 Boulevard Laurier, S-763, Quebec City, QC, Canada G1V 4G2

## Abstract

Though vitamin D is important for bone health, little is known about the monitoring and management of vitamin D levels in patients with osteoporosis in clinical practice—a deficit this chart review initiative aimed to remedy. A total of 52 physicians completed profiles for 983 patients being treated for osteoporosis between November 2008 and April 2009. Information collected included demographics; fracture risk factors; availability and level of serum vitamin D measurements; and information on osteoporosis medications and calcium and vitamin D supplementation. Physicians also evaluated patients' current regimens and detailed proposed changes, if applicable. Nearly 85% of patients were prescribed calcium and vitamin D supplements. Serum 25-hydroxy vitamin D levels were available for 73% of patients. Of these patients, approximately 50% had levels less than 80 nmol/L, which contrasts with the 37% thought to have “unsatisfactory” vitamin D levels based on physician perceptions. Physicians felt 26% of patients would benefit from additional vitamin D supplementation. However, no changes to the osteoporosis regimen were suggested for 48% of patients perceived to have “unsatisfactory” vitamin D levels. The results underscore the importance of considering vitamin D status when looking to optimize bone health.

## 1. Introduction

The estimated lifetime risk of osteoporosis is approximately 50% in women and 20% in men, making it one of the most commonly encountered conditions in primary care [1]. Fragility fractures due to osteoporosis are associated with an increased risk of death. The risk of death during the first year following a hip fracture is increased 3.17 times (95% confidence interval [CI] 1.35, 7.42), while the risk of death during the second year following vertebral fracture is increased 2.71 times (95% CI 1.12, 6.57) [2].

Vitamin D plays a critical role in maintaining bone health and preventing fragility fractures [3–7]. Lower serum vitamin D levels have been shown to be associated with decreased bone mineral density of the total hip and hip trochanter, increased risk of hip fracture in older men and women, and reduced response to bisphosphonate treatment in postmenopausal women with osteoporosis [6–9]. Conversely, though a recent meta-analysis suggests there is no clear association between vitamin D supplementation and bone mineral density in patients without vitamin D deficiency [10], vitamin D supplementation has been shown to reduce the risk of nonvertebral and hip fracture in a dose-dependent manner [11]. In accordance with these findings, it has been suggested that vitamin D may work through bone density-independent mechanisms to improve bone health [12]. Supplementation with vitamin D to increase serum 25-hydroxy vitamin D (25[OH]D) levels is thus considered an integral part of osteoporosis therapy [13–16].

Despite the importance of vitamin D for bone health and recommendations for supplementation, there is some evidence to suggest vitamin D levels are frequently lower than the 75 nmol/L recommended in patients with osteoporosis by the Osteoporosis Canada and American Association of Clinical Endocrinologists guidelines. For instance, in a study of 1536 North American women receiving osteoporosis therapy, serum 25(OH)D was less than 75 nmol/L in nearly half the subjects [17]. However, there is limited information regarding how these data translate into clinical practice.

This chart review initiative was undertaken to better understand how vitamin D levels are assessed and managed in Canadian patients with osteoporosis.

## 2. Materials and Methods

Canadian physicians involved in the management of osteoporosis were invited by fax and e-mail to take part in this initiative. Invitations were sent to 166 specialists, mainly rheumatologists, and 582 primary care practitioners from the Canadian provinces of Ontario and Quebec. The physicians who accepted this invitation used either a secure online tool or paper forms to complete a practice profile questionnaire and profiles for each of approximately 20 patients in their practice who were being treated to prevent fractures and whom they had last seen between November 2008 and April 2009 (see additional file 1, practice profile form, and additional file 2, patient profile form in Supplementary Material available online at http://dx.doi.org/10.1155/2015/312952). Information collected in the patient profiles included demographic data, bone mineral density (BMD) and other fracture risk factor information, availability and level of serum vitamin D measurements, current osteoporosis medications, and levels of calcium and vitamin D supplementation. Participants were also asked to evaluate patients' current regimens and detail any proposed changes. In order to maintain confidentiality, no identifying information (e.g., name, birth date, and postal code) was collected.

Bivariate generalized estimating equations (GEE) were used to investigate factors potentially associated with physician perception of “satisfactory” vitamin D levels and with suggested addition or dose increases of vitamin D supplementation. All variables associated with a *P* value of <0.2 in the bivariate analyses were then included in multivariable GEE analyses. The GEE technique utilized an exchangeable correlation structure. The GEE method was conducted to factor in the clustered nature of the data given that individuals managed by the same physician will be treated similarly. Adjusted odds ratios and 95% confidence intervals (CI) were calculated. All analyses were performed with SAS statistical software version 9.2 for Windows (SAS, North Carolina).

This chart review initiative was designed and reviewed by the authors to ensure compliance with the World Medical Association Declaration of Helsinki regarding ethical principles for medical research. Given the retrospective design and anonymous nature of the data collected, no approval from an official ethical review board was sought.

## 3. Results

### 3.1. Participating Physicians

Of the 582 primary care practitioners invited to take part in this initiative, 36 primary care physicians agreed to participate, for a response rate of 6.4%. Similarly, 16 (9.6%) specialists of the 166 who were invited participated. A total of 36 physicians (28 primary care practitioners and 8 specialists) were from Ontario, while the remaining 16 (8 primary care practitioners and 8 specialists) were from Quebec. Most (78.8%, *n*/*N* = 41/52) of the participating physicians worked in an urban area and just over half (55.8%, *n*/*N* = 29/52) were in solo rather than a group practice. Approximately 80% (*n*/*N* = 42/52) had been practicing for over 20 years. The number of patient cases related to osteoporosis seen by the physicians each week varied greatly, with 30.8% (*n*/*N* = 16/52) seeing 10 or fewer cases and 23.1% (*n* = 12/52) seeing over 20 cases each week.

### 3.2. Patient Characteristics

Profiles were completed for a total of 983 patients who were being treated for osteoporosis and who were last seen by participating physicians between November 2008 and April 2009. As shown in Table 1, over 85% (*n*/*N* = 863/983) of patients were female and 64% (*n*/*N* = 626/983) were 60 to 79 years of age. Mean bone mineral density (BMD) and the standard deviation (SD) based on most recent lowest T-score were −2.16 (1.55). Over a quarter of patients (*n*/*N* = 272/983) had had a previous fragility fracture, including 38% (*n*/*N* = 57/151) of those with BMD ≥ −1.0 and 27% (*n*/*N* = 151/563) of those with BMD ≥ −2.5.

**Figure 1:**
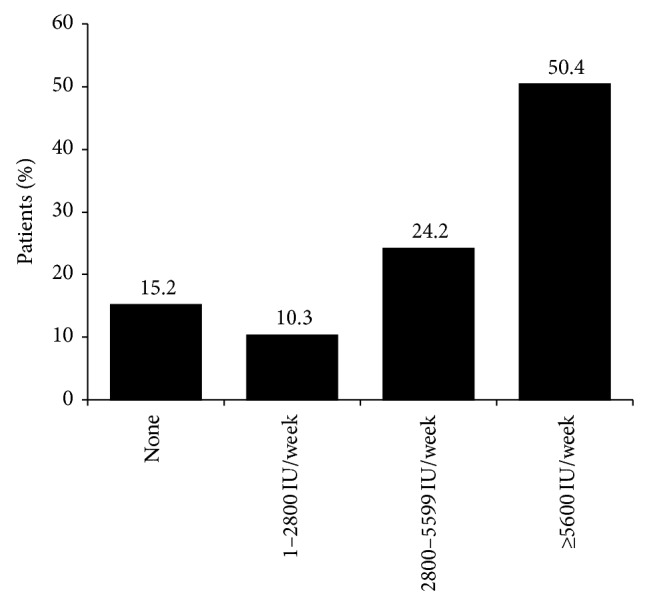
Level of vitamin D supplementation (*n* = 983). Note: numbers may not add to 100 due to rounding.

Almost all patients had been receiving treatment for osteoporosis for over a year, as shown in Table 1. Bisphosphonates, prescribed to nearly 80% (*n*/*N* = 777/983) of patients, were the most commonly used pharmacotherapy and 85% (*n*/*N* = 834/983) of patients were prescribed calcium and vitamin D supplements. Most patients (77.2%, *n*/*N* = 759/983) received more than 600 mg/day of supplemental calcium, as seen in Figure 1. As shown in Figure 1, approximately half (*n*/*N* = 495/983) of patients were prescribed ≥5600 IU per week of vitamin D supplementation, in accordance with Osteoporosis Canada recommendations [11].

### 3.3. Serum 25(OH)D Levels

**Figure 2:**
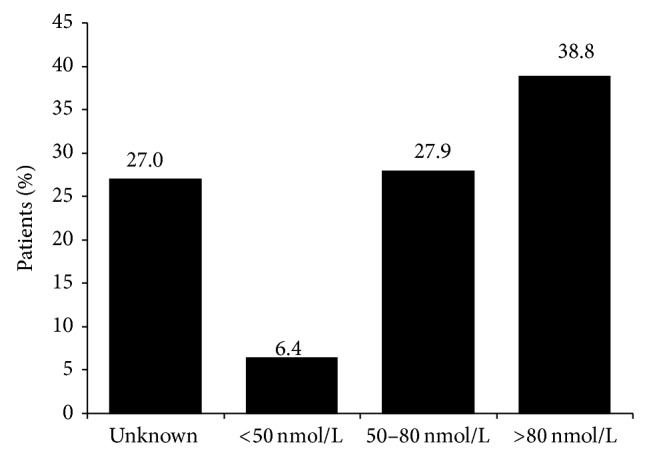
Serum 25(OH)D levels (*n* = 983). Note: numbers may not add to 100 due to rounding.

**Figure 3:**
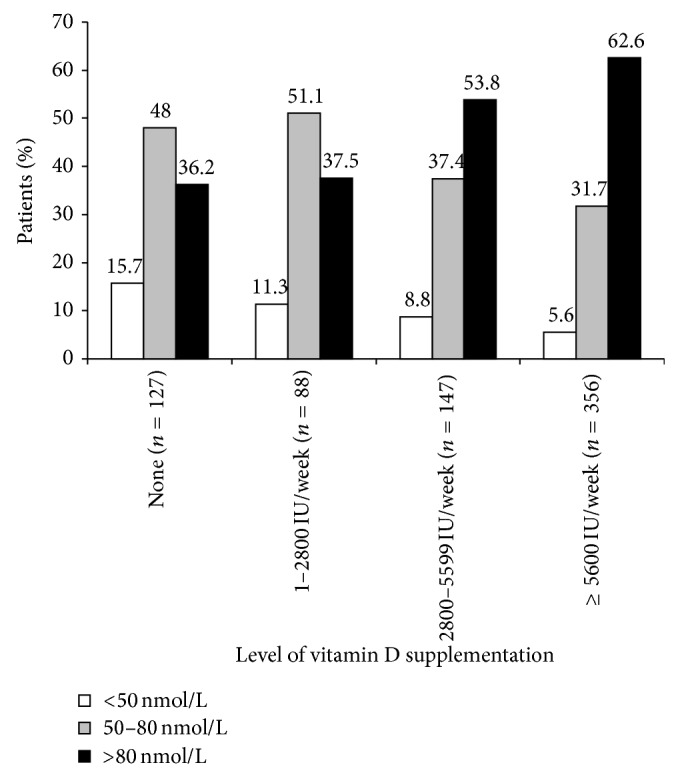
Serum 25(OH)D levels by prescribed level of vitamin D supplementation (*n* = 718). Note: numbers may not add to 100 due to rounding.

Serum 25(OH)D levels were available in charts for 61.2% (*n*/*N* = 602/983) of patients and were measured during the course of the initiative for a further 11.8% (*n*/*N* = 116/983) of patients, meaning serum 25(OH)D levels were available for 73.0% (*n*/*N* = 718/983) of patients. In those patients for whom measurements were available, the mean serum 25(OH)D level (SD) was 85.0 (29.0) nmol/L and approximately half of these patients had levels less than 80 nmol/L (see Figure 2). Over 60% (*n*/*N* = 136/215) of patients prescribed less than 2800 IU/week of vitamin D had serum 25(OH)D levels less than 80 nmol/L, as compared to 46% (*n*/*N* = 68/147) and 37% (*n*/*N* = 133/356) of those prescribed 2800–5599 IU/week and at least 5600 IU/week, respectively (see Figure 3).

### 3.4. Physician Perceptions of Management and Changes to Osteoporosis Regimen

**Figure 4:**
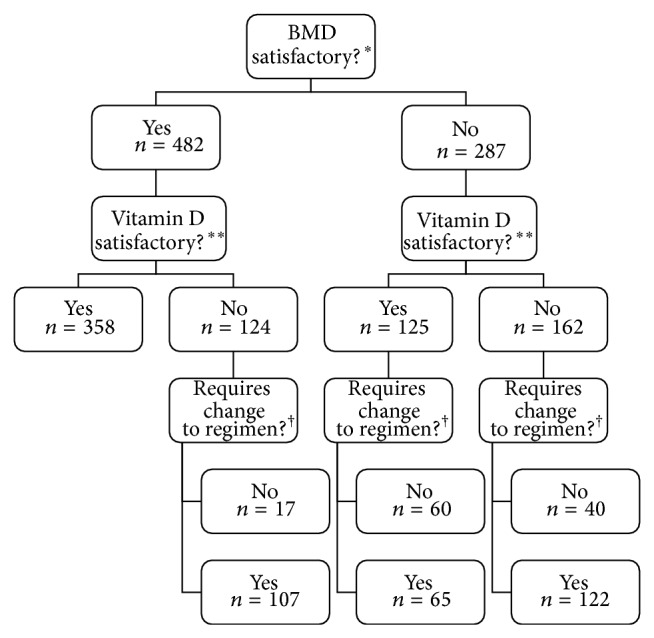
Physician evaluation of osteoporosis regimens. ^*^Based on answers to the question, “Is this patient's BMD satisfactory (i.e., has there been no significant decrease in any measurement between the most recent and previous scans)?” (BMD = bone mineral density). ^**^Based on answers to the question, “Are this patient's serum vitamin D levels adequate?” ^†^Based on answers to the question, “If you answered ‘No' to one of the above questions, does this patient require any changes to their medication?”

Participating physicians evaluated the osteoporosis treatment regimens for 769 patients, 37% (*n*/*N* = 287/769) of whom were considered to have “unsatisfactory” change in BMD and 37% (*n*/*N* = 286/769) of whom were considered to have “unsatisfactory” vitamin D levels (see Figure 4). Just over half (*n*/*N* = 162/287) of patients considered to have “unsatisfactory” changes in BMD were also considered to have “unsatisfactory” vitamin D levels, as compared to 25% (*n*/*N* = 124/482) of those with “satisfactory” changes in BMD. As shown in Table 2, multivariable GEE analysis suggests that serum 25(OH)D level and body mass index were the only factors significantly associated with physicians' perceptions of whether vitamin D levels were satisfactory.

Of the patients who were considered by physicians to have “unsatisfactory” changes in BMD or vitamin D levels or both, about 70% (*n*/*N* = 294/411) were thought to require changes to their osteoporosis regimen (see Figure 4). Additional vitamin D supplementation was the most common change suggested, occurring in 78.3% (*n*/*N* = 224/286 patients) of these patients. Physicians did not recommend increases in vitamin D supplementation in 46.9% (*n*/*N* = 158/337) of patients with known serum 25(OH)D less than 80 nmol/L. Based on multivariable GEE analyses, suggested changes to vitamin D supplementation were significantly associated with a perception of unsatisfactory vitamin D levels. No other variable was found to be significant (see Table 3). Other suggested changes to osteoporosis therapy for the 294 thought to require changes included counselling regarding lifestyle modifications (40.8%), additional calcium supplementation (30.6%), changing medication to a bisphosphonate (21.4%), and increasing the dose of current medication (13.6%). In addition, 62.8% of the 385 patients for whom a question about combination therapy was answered were felt to possibly benefit from combination therapy with vitamin D.

## 4. Discussion

This chart review initiative found that a significant proportion of patients treated for osteoporosis in clinical practice have insufficient vitamin D levels, as approximately half of those with known serum 25(OH)D levels had a level less than 80 nmol/L, in many cases despite supplementation with at least 5600 IU of vitamin D per week. In addition, the facts that physicians did not recommend increases in vitamin D supplementation in nearly half of patients with serum 25(OH)D levels less than 80 nmol/L and that serum 25(OH)D level was not significantly associated with changes to vitamin D supplementation suggest that management of vitamin D levels may be a potential gap in practice.

This finding is not surprising as, until recently, there has been little guidance for Canadian physicians regarding how vitamin D levels should be monitored and measured in clinical practice. Osteoporosis Canada guidelines published in the fall of 2010 recommend serum 25(OH)D levels be measured in individuals with osteoporosis after three or four months of receiving adequate vitamin D supplementation to ensure levels are ≥75 nmol/L [13, 14]. However, earlier Canadian osteoporosis recommendations did not provide guidance regarding whether vitamin D levels should be measured or what levels should be considered optimal [18, 19]. Conversely, more recently, several publications have suggested cut-off serum 25(OH)D levels of as low as 50 nmol/L might be adequate to promote optimal bone health [16, 20], though the 2011 US Institute of Medicine recommendations also cite >75 nmol/L as the optimal level [21]. In this regard, it should be noted that the 80 nmol/L threshold for vitamin D sufficiency used in this initiative reflects Canadian reference values used prior to the publication of the Osteoporosis Canada guidelines.

The relatively high levels of vitamin D insufficiency (serum 25[OH]D less than 80 nmol/L) seen in patients prescribed vitamin D supplementation may indicate that adherence to supplementation may be an issue for many patients. In fact, adherence to calcium and vitamin D supplements has been shown to be significantly poorer than for pharmacologic osteoporosis therapies, such as bisphosphonates [22]. In an Italian survey of 9851 postmenopausal women with osteoporosis, approximately half of women prescribed supplements took less than 80% of the prescribed supplementation pills, while about 20% of patients took less than half of the required pills and one in five patients discontinued use of supplementation within one year [22]. Such nonadherence might be one explanation for some of the low serum 25(OD) levels seen in patients prescribed supplementation. Comorbid conditions affecting vitamin D absorption could be another explanation. For instance, obese individuals often suffer from vitamin D inadequacy [23, 24], likely due to sequestration of fat-soluble vitamin D in body fat compartments and a resulting decrease in bioavailable vitamin D [23]. In this context it is interesting that BMI was one of the two factors significantly associated with physician perception of vitamin D status in this initiative. However, no data on other conditions known to impact vitamin D absorption, such as intestinal malabsorption syndrome [25], were captured in the chart review process.

Other limitations of this study include the fact that participation was restricted to physicians in Ontario and Quebec and the low response rate. In addition, participating physicians were not instructed to select consecutive patients for chart review, thus creating the potential for patients to be selectively chosen. Indeed, the high proportion of patients with serum 25(OH)D levels available in file may indicate a selection bias either towards physicians with a greater than average interest in monitoring vitamin D levels or towards patients considered at risk for low vitamin D levels in whom serum 25(OH)D might be more likely to be measured.

## 5. Conclusions

Although most physicians recommend calcium and vitamin D supplementation, vitamin D levels appear to be insufficient in half of patients, including over 40% of patients prescribed at least 2800 IU per week of vitamin D supplementation. This, when added to the fact that physicians did not recommend increases in vitamin D supplementation in nearly half of patients with known serum 25(OH)D less than 80 nmol/L, suggests a potential gap in practice. Recent changes to the Canadian osteoporosis guidelines clarifying when to measure serum vitamin D and what levels should be considered optimal may help promote monitoring of serum vitamin D status and adequate vitamin D supplementation. However, underlying causes of vitamin D insufficiency, including comorbidities and nonadherence, will also need to be addressed. The results underscore the importance of considering vitamin D status when looking to optimize bone health.

## Supplementary Material

Characterizing the assessment and management of vitamin D levels in patients with osteoporosis in clinical practice: a chart review initiative
The following two questionnaires were used for this chart review initiative aimed to better understand how vitamin D levels are assessed and managed in Canadian patients with osteoporosis.
The practice profile form includes questions on the physician's demographic, years in practice, practice settings and continuing medical education interests.
The patient profile form includes questions on demographic, bone mineral density (BMD) and other fracture risk factor information, availability and level of serum vitamin D measurements, current osteoporosis medications, levels of calcium and vitamin D supplementation and current regiment and proposed changes.

## Figures and Tables

**Table 1:** Patient characteristics.

Characteristic	Percentage^*^	Sample size
Gender (female)	87.8%	983
Age		983
<50 years	2.0%	
50–59 years	14.5%	
60–69 years	30.0%	
70–79 years	33.7%	
≥80 years	19.7%	
BMD based on most recent lowest *T*-score, mean (SD)	−2.16 (1.55)	946
Additional fracture risk factors		977
Previous fragility fracture	27.8%	
Systemic glucocorticoids for >3 months	8.5%	
Previous fragility fracture and systemic glucocorticoids for >3 months	3.3%	
Length of treatment for osteoporosis		980
<1 year	11.3%	
1–5 years	43.8%	
>5 years	44.9%	
Osteoporosis therapy		983
Bisphosphonate	79.0%	
Selective estrogen-receptor modulator	3.0%	
Hormone therapy	2.3%	
Parathyroid hormone	2.2%	
Calcitonin	1.3%	
Prescribed supplementation		983
Calcium	84.8%	
Vitamin D	84.8%	

^*^Except for BMD, for which the mean (SD) is presented.

BMD = bone mineral density; SD = standard deviation.

**Table 2:** Results of multivariable GEE analysis examining potential associations with perception of “satisfactory” vitamin D levels.

Parameter	OR (95% CI)
Treated by a physician practicing for ≤20 years	2.34 (0.67, 8.19)
Treated by a physician seeing ≤10 patients with osteoporosis per week	1.78 (0.69, 5.38)
No current vitamin D supplementation	1.44 (0.86, 2.40)
Serum 25(OH)D level	1.10 (1.04, 1.17)
Body mass index	1.05 (1.00, 1.09)
Age	1.01 (0.99, 1.03)
Living in Ontario	0.51 (0.16, 1.68)

25(OH)D = 25-hydroxy vitamin D; CI = confidence interval; GEE = generalized estimating equation; OR = odds ratio.

**Table 3:** Results of multivariable GEE analysis examining potential associations with change in vitamin D supplementation.

Parameter	OR (95% CI)
Vitamin D level believed to be unsatisfactory	35.70 (12.69, 100.43)
Living in Ontario	2.32 (0.70, 7.64)
Treated for osteoporosis for less than 1 year	1.69 (0.62, 4.59)
No current therapy for osteoporosis	1.37 (0.81, 2.31)
Treated for osteoporosis for 1–5 years	1.31 (0.74, 2.33)
Decrease in BMD	1.29 (0.81, 2.04)
Serum 25(OH)D level	1.00 (0.98, 1.01)
Body mass index	0.99 (0.95, 1.02)
Treated by a general practitioner or family physician	0.65 (0.30, 1.39)

25(OH)D = 25-hydroxy vitamin D; BMD = bone mineral density; CI = confidence interval; GEE = generalized estimating equation; OR = odds ratio.
