# Supplementary material for: Characterizing the Assessment and Management of Vitamin D Levels in Patients with Osteoporosis in Clinical Practice: A Chart Review Initiative
Source: J Osteoporos. 2015 Jan 29;2015:312952. doi: 10.1155/2015/312952 (PMC4325965; doi:10.1155/2015/312952)
Supplement: Supplementary file 1 — Characterizing the assessment and management of vitamin D levels in patients with osteoporosis in clinical practice: a chart review initiative The following two questionnaires were used for this chart review initiative aimed to better understand how vitamin D levels are assessed and managed in Canadian patients with osteoporosis. The practice profile form includes questions on the physician's demographic, years in practice, practice settings and continuing medical education interests. The patient profile form includes questions on demographic, bone mineral density (BMD) and other fracture risk factor information, availability and level of serum vitamin D measurements, current osteoporosis medications, levels of calcium and vitamin D supplementation and current regiment and proposed changes. [file 312952.f1.pdf]

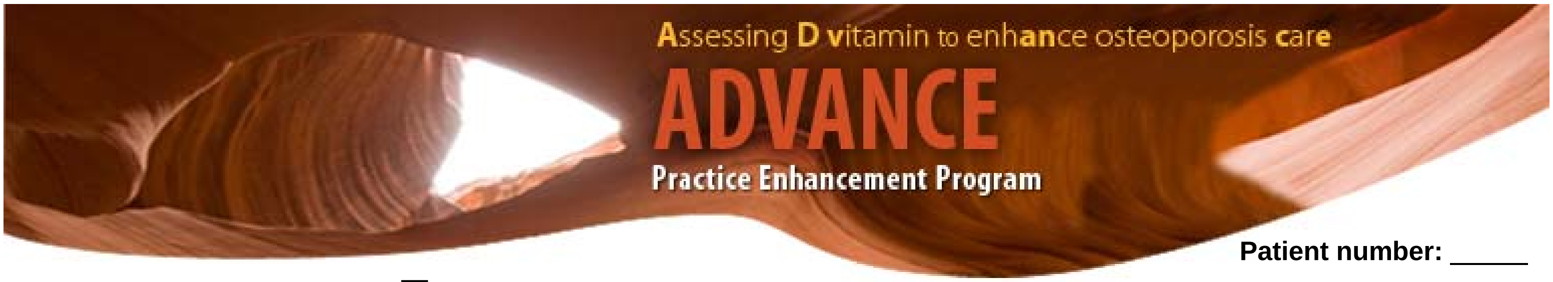

Patient number: _____

Inclusion Criteria: ☐ Treated to prevent fracture and last seen between November 2008 and April 2009

Patient Information:

Gender: ☐ Male ☐Female **Age:** _______ years **Height:** _______ cm **Weight**: _______ kg

Race: ☐ Asian ☐ Black ☐ Caucasian ☐ Other

Length of treatment to prevent fracture: ☐ <1 year ☐ 1-5 years ☐ >5 years

Fragility fracture after age 40: ☐ Spine ☐ Hip ☐ Wrist ☐ Other: ___________________________ ☐ None

Systemic glucocorticoid therapy of >3 months' duration: ☐ Yes ☐ No

Most recent bone mineral density (BMD) measurement (lowest T-score): ________________________________

Date of most recent BMD measurement: ____

Site of lowest T-score: ☐ Lumbar spine ☐ Total hip ☐ Femoral neck ☐ Trochanter ☐ Other: __________

Significant change in BMD between most recent and previous scans:*[1]

Lumbar spine	☐ Increase	☐ Decrease	☐ None	☐ N/A
Total hip	☐ Increase	☐ Decrease	☐ None	☐ N/A
Femoral neck	☐ Increase	☐ Decrease	☐ None	☐ N/A
Trochanter	☐ Increase	☐ Decrease	☐ None	☐ N/A

Serum vitamin D availability: ☐ Available in chart ☐ Test had to be scheduled → Date scheduled: _________

Serum vitamin D level (nmol/L): __________________ Month measurement taken: _______________________

If serum vitamin D level is not available, please consider arranging to have patient tested for serum 25(OH)D level (assay L606 on schedule of laboratory fees[2]). Note: there is no charge to the patient for this assay.

Current Osteoporosis Medications

None	☐		
Bisphosphonate			
Alendronate (Fosamax®)	☐ 5 mg/day	☐ 10 mg/day	☐ 70 mg/week
Alendronate/cholecalciferol (Fosavance®)	☐ 70 mg/2800 IU per week	☐ 70 mg/5600 IU per week	
Etidronate/calcium carbonate (Didrocal®)	☐ 400 mg/1250 mg		
Risedronate (Actonel®)	☐ 5 mg/day	☐ 35 mg/week	☐ 150 mg/month
Risedronate – calcium carbonate (Actonel® Plus Calcium)	☐ 35 mg/week – 1250 mg/day (six days/week)		
Zoledronic acid (Aclasta)	☐ 5 mg/100 mL yearly infusion		
Hormone therapy			
Estradiol-17β (Climara®, Estraderm®, Estradot®)	☐ 25 μg/day	☐ 37.5 μg/day<br>☐ 75 μg/day	☐ 50 μg/day<br>☐ 100 μg/day
Estradiol-17β (micronized) (Estrace®)	☐ 0.5 mg/day	☐ 1 mg/day	☐ 2 mg/day
Estradiol-17β – norethindrone acetate/estradiol-17β (Estracomb®)	☐ 50 μg/day – 250 μg/50 μg per day		
Norethindrone acetate/ethinyl estradiol (femHRT™)	☐ 0.5 mg/2.5 μg per day	☐ 1 mg/5 μg per day	
Parathyroid hormone			
Teriparatide (Forteo®)	☐ 20 μg/day		
Calcitonin			
Salmon calcitonin (Miacalcin® NS)	☐ 200 IU/day		
Selective estrogen receptor modulator			
Raloxifene (Evista®)	☐ 60 mg/day		
Supplementation			
Calcium supplement	☐ None<br>☐ 601-1199 mg/day	☐ <500 mg/day<br>☐ ≥1200 mg/day	☐ 500-600 mg/day
Vitamin D supplement	☐ None<br>☐ 2800-5599 IU/week	☐ <2800 IU/week<br>☐ ≥5600 IU/week	
Other (please specify): ______________________________		Dose: ______________________	

N/A = not applicable

*The least significant change between two BMD measurements is 2.8 times the site-specific precision error for a single measurement.[1] The patient's most recent BMD report should note whether a significant change in BMD was observed at any of the sites.

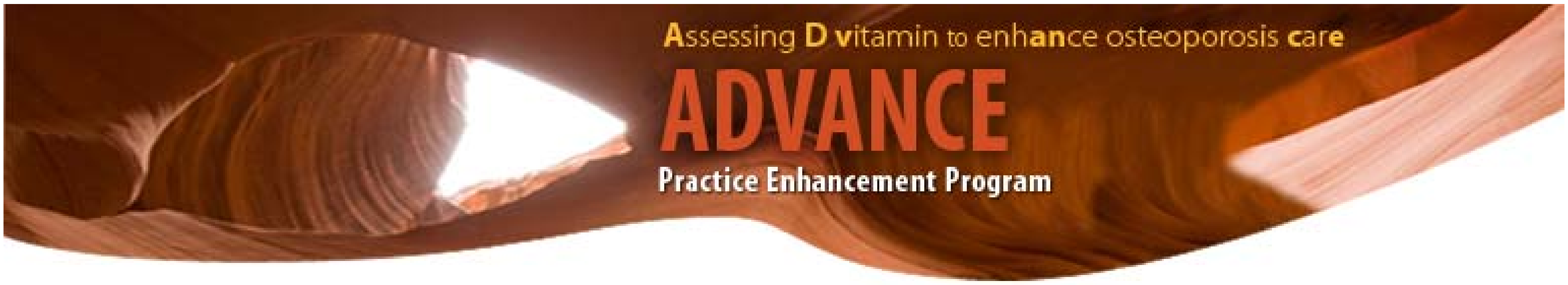

Evaluation of Current Regimen

Is this patient's BMD satisfactory (i.e., has there been no significant decrease in any measurement between the most recent and previous scans)?[3] ☐ Yes ☐ No

Are this patient's serum vitamin D levels adequate? ☐ Yes ☐ No

Implications of Various Serum Vitamin D Levels[4]	
<50 nmol/L	Deficiency
50-79 nmol/L	Insufficiency
80-250 nmol/L	Sufficiency

If you answered "No" to one of the above questions, does this patient require any changes to their medication? ☐ Yes ☐ No

If patient requires changes to medication, please complete the section below and consider arranging an appointment in order to implement proposed changes.

Changes to Regimen (if applicable)

Please indicate what changes you intend to make, if applicable (please check all that apply):

☐ **A.** Increase dose of current medication	☐ **B.** Change to another medication:	☐ **C.** Add or increase dose of supplement:	☐ **D.** Provide counselling regarding lifestyle modifications
	☐ Bisphosphonate<br>☐ Parathyroid hormone<br>☐ Calcitonin<br>☐ Hormone therapy<br>☐ Selective estrogen receptor modulator	☐ Calcium<br>☐ Vitamin D	

Would this patient benefit from combination therapy with vitamin D? ☐ Yes ☐ No

Please check medication(s) and supplement(s) to be included in proposed regimen:

Bisphosphonate			
Alendronate (Fosamax®)	☐ 5 mg/day	☐ 10 mg/day	☐ 70 mg/week
Alendronate/cholecalciferol (Fosavance®)	☐ 70 mg/2800 IU per week	☐ 70 mg/5600 IU per week	
Etidronate/calcium carbonate (Didrocal®)	☐ 400 mg/1250 mg		
Risedronate (Actonel®)	☐ 5 mg/day	☐ 35 mg/week	☐ 150 mg/month
Risedronate – calcium carbonate (Actonel® Plus Calcium)	☐ 35 mg/week – 1250 mg/day (six days/week)		
Zoledronic acid (Aclasta)	☐ 5 mg/100 mL yearly infusion		
Hormone therapy			
Estradiol-17β (Climara®, Estraderm®, Estradot®)	☐ 25 µg/day	☐ 37.5 µg/day<br>☐ 75 µg/day	☐ 50 µg/day<br>☐ 100 µg/day
Estradiol-17β (micronized) (Estrace®)	☐ 0.5 mg/day	☐ 1 mg/day	☐ 2 mg/day
Estradiol-17β – norethindrone acetate/estradiol-17β (Estracomb®)	☐ 50 µg/day – 250 µg/50 µg per day		
Norethindrone acetate/ethinyl estradiol (femHRT™)	☐ 0.5 mg/2.5 µg per day	☐ 1 mg/5 µg per day	
Parathyroid hormone			
Teriparatide (Forteo®)	☐ 20 µg/day		
Calcitonin			
Salmon calcitonin (Miacalcin® NS)	☐ 200 IU/day		
Selective estrogen receptor modulator			
Raloxifene (Evista®)	☐ 60 mg/day		
Supplementation			
Calcium supplement	☐ None<br>☐ 601-1199 mg/day	☐ <500 mg/day<br>☐ >1200 mg/day	☐ 500-600 mg/day
Vitamin D supplement	☐ None<br>☐ 2800-5599 IU/week	☐ <2800 IU/week<br>☐ >5600 IU/week	
Other (please specify): ______________________		Dose: ______________________	

Follow-up appointment scheduled: ☐ Yes ☐ No

1. Siminoski K et al. Can Assoc Radiol J 2005; 56:178-88.
2. Ontario Ministry of Health and Long-Term Care. Schedule of Benefits for Laboratory Services [Online]. 2007. [cited 2008 October 23]. Available from: http://www.health.gov.on.ca/english/providers/program/ohip/sob/lab/lab_ services_ sched _01_19990401.pdf.
3. International Society for Clinical Densitometry. Canadian Physician Pocket Guide to BMD Testing [Online]. 2004 [cited 2008 May 26]. Available from: http://www.iscd.org/Visitors/pdfs/ISCD-CANADIANPanelOfficialPositions-BMDcard.pdf.
4. Grant WB, Holick MF. Altern Med Rev 2005; 10:94-111.
